# ESC-NAS: Environment Sound Classification Using Hardware-Aware Neural Architecture Search for the Edge

**DOI:** 10.3390/s24123749

**Published:** 2024-06-09

**Authors:** Dakshina Ranmal, Piumini Ranasinghe, Thivindu Paranayapa, Dulani Meedeniya, Charith Perera

**Affiliations:** 1 Department of Computer Science & Engineering, University of Moratuwa, Moratuwa 10400, Sri Lanka; dakshina.19@cse.mrt.ac.lk (D.R.); piumini.19@cse.mrt.ac.lk (P.R.); thivindu.19@cse.mrt.ac.lk (T.P.); dulanim@cse.mrt.ac.lk (D.M.); 2 School of Computer Science and Informatics, Cardiff University, Cardiff CF24 3AA, UK

**Keywords:** deep learning, environment sound classification, hardware-aware neural architecture search, lightweight convolutional neural networks, search space

## Abstract

The combination of deep-learning and IoT plays a significant role in modern smart solutions, providing the capability of handling task-specific real-time offline operations with improved accuracy and minimised resource consumption. This study provides a novel hardware-aware neural architecture search approach called ESC-NAS, to design and develop deep convolutional neural network architectures specifically tailored for handling raw audio inputs in environmental sound classification applications under limited computational resources. The ESC-NAS process consists of a novel cell-based neural architecture search space built with 2D convolution, batch normalization, and max pooling layers, and capable of extracting features from raw audio. A black-box Bayesian optimization search strategy explores the search space and the resulting model architectures are evaluated through hardware simulation. The models obtained from the ESC-NAS process achieved the optimal trade-off between model performance and resource consumption compared to the existing literature. The ESC-NAS models achieved accuracies of 85.78%, 81.25%, 96.25%, and 81.0% for the FSC22, UrbanSound8K, ESC-10, and ESC-50 datasets, respectively, with optimal model sizes and parameter counts for edge deployment.

## 1. Introduction

Deep learning (DL) exhibits promising results, as it can automatically extract features from data, while handling massive amounts of complex data formats in different application domains [1]. DL-based smart surveillance systems can use various data modalities, including sound, video, and sensor data, to gain insights. Out of these different input types, sound data can be a valuable source of information for surveillance systems, which can be used to detect suspicious activities, identify individuals, and track their movements [2,3]. Accounting for these aspects, this study focuses on environmental sound classification using DL on resource-constrained IoT devices.

Environmental sound classification plays a pivotal role in identifying, analyzing, managing, and interacting across different rural and urban domains, and thereby enhancing safety, sustainability, and quality of life. Deep learning (DL)-based smart surveillance finds applications across diverse domains of environmental sound classification, including smart homes, industrial surveillance, traffic monitoring, and environmental monitoring [1,3]. If further dived into applications in environmental monitoring, it proves instrumental in wildlife tracking, animal behavior analysis, and the detection of illicit activities such as logging and poaching. Many surveillance applications necessitate real-time responsiveness, a challenge for which DL exhibits potential. DL can facilitate intelligent and automated systems capable of real-time data analysis and extraction of valuable insights [4].

The confluence of DL and the IoT presents substantial potential for enhancing the efficiency and optimality of artificial intelligence (AI) applications, surpassing conventional signal processing and traditional machine learning approaches [5]. IoT devices, leveraging sensor data acquisition and generation, synergize with the capabilities of DL models. The amalgamation of these technologies leads to the development of intelligent, interconnected systems, fostering heightened accuracy and efficiency. This synergy further facilitates automated, real-time decision-making and enhances adaptability within the framework of AI applications. Edge devices often have limited computing power, memory, and battery life. Due to such resource limitations, running complex DL models on resource-constrained edge devices is challenging. This study proposes a neural architecture search (NAS)-based approach for classifying raw environmental sounds that can be deployed in resource-constrained edge devices.

NAS is a rapidly evolving field in DL that focuses on automating the design of artificial neural networks (ANNs). Traditionally, the design of ANN architectures was mainly dependent on human expertise, intuition, and a trial-and-error approach. NAS seeks to revolutionize this manual process by introducing automated systems that systematically explore an extensive space of potential architectures, to identify the optimal DL architecture for a given classification [6]. NAS techniques provide significant accuracy and robustness benefits when coming up with convolutional neural network (CNN) architectures to solve a wide range of problems. This is due to the intractable design space, nontransferable optimality, and inconsistent efficiency matrices [7]. NAS has the potential to discover novel and highly optimized architectures that outperform human-designed models [8]. NAS significantly accelerates the process of designing DL models for classifying raw audio inputs using edge devices in resource-constrained environments.

Since this study’s targeted model deployment environment comprises resource-constrained edge devices, solely relying on NAS would be a limitation. Hardware-aware NAS (HW-NAS), which specifically focuses on required resource constraints while searching for optimal architectures, becomes necessary. Among the existing literature that followed different approaches for narrowing down the NAS process to adhere to given limitations [9,10,11,12], this study uses hardware simulation to estimate computational costs such as RAM and memory usage. Another notable aspect of this study is exploring an optimal HW-NAS model for raw audio classification on resource-constrained edge devices. Most of the DL models used in audio classification incorporate feature extraction techniques such as Mel-spectrograms, short-time Fourier transform (STFT), and Mel-frequency cepstral coefficients (MFCC) [3]. Although these techniques extract meaningful information from raw audio, the employment of feature extraction techniques proves resource-intensive in resource-constrained environments, thereby reducing the resources available to improve model performance. Moreover, feature extraction from input audio introduces latency in real-time inferences. For applications such as smart surveillance where timely inferences are crucial, minimizing such delays is imperative to enhance the overall system performance. Hence, this study focuses on designing an optimal DL model that takes raw audio as the input and leverages the capabilities of CNNs to simultaneously extract features and identify the given audio clip.

Several studies have used NAS for audio classification in many use cases, such as music instrument recognition, speech recognition, keyword spotting, and audio tagging [13,14,15]. However, the employment of NAS is limited within the domain of environmental sound classification. Moreover, in the existing audio classification studies employing NAS, feature extraction techniques such as Mel and MFCC were commonly utilized [16,17], akin to those in image classification tasks. Addressing the aforementioned research gaps, this study endeavored to pursue the following research questions:

- RQ1: With the general nature of DL models being highly resource intensive, how can one design a DL model for environmental sound classification in resource-constrained environments?
- RQ2: How can HW-NAS be used as a potential approach to automate the designing of DL models with awareness of computational restrictions for environmental sound classification on resource-constrained edge devices?
- RQ3: How can a search space be defined for the HW-NAS process, comprising appropriate DL layers that, when combined, can construct an optimal DL model capable of classifying raw audio inputs without relying on feature extraction techniques?

Henceforth, the novelty of this study lies in its novel approach of employing HW-NAS in raw audio classification as a case study for environmental sound classification. Although there are a limited number of current literature works on audio classification using HW-NAS [12,18,19,20], there are no literature studies, to the best of our knowledge, that performed HW-NAS taking raw audio as the input for the DL model. In order to design such a model, we introduce a novel search space for the proposed HW-NAS process, named ESC-NAS. This novel search space consists of a series of 2D convolution layers, batch normalization layers, rectified linear unit (ReLU) activation function layers, and max pooling operation layers. Additionally, we prove the compatibility of the ESC-NAS process across four publicly available environmental sound classification datasets, while obtaining better trade-offs between model performance and resource consumption when compared to the existing literature. This study further confirmed the performance of the resulting optimal architecture through deployment on a Raspberry Pi 3 model B+ and evaluating memory utilization and inference latency.

The structure of this paper is as follows: Section 2 provides background information on NAS and explores the related existing literature. Section 3 explains how the study was conducted. Section 4 presents the results obtained. Section 5 discusses the results, lessons learned, and future research directions. Finally, the paper is concluded with Section 6.

## 2. Background

### 2.1. Environment Sound Classification

Environment sound classification (ESC), which leverages machine learning approaches to analyze and categorize sounds within an audio recording, has emerged as a powerful tool for applications across various domains, such as smart homes, audio surveillance, robot navigation, security surveillance, wildlife monitoring, forest monitoring, and noise monitoring [21]. Moreover, ESC holds significance due to its provision of a non-invasive and objective approach for analyzing soundscapes, thereby offering several advantages in surveillance applications. To realize the aforementioned benefits of ESC, it must demonstrate high sound recognition accuracy and be deployable on IoT devices. Achieving high accuracy in sound recognition often necessitates the utilization of DL techniques, consistently with prevailing trends across various ML applications [22]. However, DL architectures are inherently complex and demand substantial computational resources, thereby constraining their deployment on the resource-constrained devices that are commonly employed in environmental surveillance applications [23,24]. This study aimed to address this challenge.

By deploying DL-based ESC models on resource-constrained edge devices, certain benefits can be achieved, including reduced latency, enhanced privacy preservation, and offline functionality. Local audio data processing on edge devices mitigates latency compared to transmission to cloud servers for analysis, which is a critical consideration for real-time applications. Additionally, sensitive audio data, such as human speech, can undergo analysis directly on the device, effectively mitigating privacy concerns typically associated with cloud storage solutions. Furthermore, edge-based ESC models can function autonomously, independently of a persistent internet connection. This capability facilitates application endeavors in remote locales, including forest environments, where connectivity may be intermittent or unavailable.

One specialized application of ESC is forest sound classification, which focuses on acoustics within forest environments. In the context of addressing the pervasive global concern about deforestation, the utilization of DL-based forest sound classification in surveillance systems has emerged as a viable mitigation strategy [3,24,25]. As traditional forest monitoring relies heavily on manual patrols and satellite imaging, which are often time-consuming, expensive, and limited in scope, DL-based forest surveillance becomes a clearly more expedient alternative.

Another key application of ESC is urban sound classification, which delves into the acoustic ecology within urban landscapes, notably cities. With advanced approaches such as DL-based audio classifiers on edge devices, urban sound classification holds immense potential for designing, managing, and monitoring sustainable urban environments that promote human well-being, and security, and can minimize noise pollution alongside ecological diversity [16,26,27].

### 2.2. Neural Architecture Search

Traditional ML pipelines consist of several steps that require human supervision to build a well-performing model. NAS is a subfield of automated machine learning (AutoML) that automates the ML pipeline. The ML pipeline encompasses data collection, data processing, feature engineering, model development, model evaluation, model deployment, and monitoring stages, where AutoML streamlines the automation of tasks spanning from data preprocessing to model evaluation within the ML pipeline. AutoML encompasses a broader scope compared to NAS, as NAS focuses specifically on automating the model development phase of the machine learning pipeline, which is the most critical component of model design.

NAS comprises three primary components: the search space, search strategy, and evaluation strategy [8]. The search spaces in NAS typically exhibit a wide breadth compared to those in hyperparameter optimization (HPO); however, exhaustively traversing these search spaces to build and evaluate all possible architectures is computationally prohibitive. Hence, an optimized search strategy is imperative for identifying the optimal architecture without necessitating an exhaustive search. Additionally, evaluation constraints, such as performance assessment on edge devices and cloud platforms, can be used to narrow down the search.

#### 2.2.1. Search Space

The search space of NAS consists of the elements to be explored during the architecture search process. It primarily comprises units or blocks that define layers, the overall structure, and permissible connectivities. The search space can enhance the efficacy of the search algorithm in discovering optimal architectures by having a diverse set of search components. However, exploring larger search spaces can significantly prolong the search process. Search spaces are typically characterized by three main representations namely, sequential layer-wise operations, chain-structured space, and cell-based representation. The sequential layer-wise operation representation involves a sequence of layers, where each layer receives the input from the preceding layer and feeds its output to the subsequent layer. In contrast, the chain-structured space offers a more complex search space, incorporating additional layer types and enabling multiple branches and skip connections. The cell-based representation utilizes components from existing models rather than constructing the search space manually, facilitating a more efficient exploration process. This study used a cell-based search space.

#### 2.2.2. Search Strategy

A search strategy is the process of identifying an optimal architecture from a search space. Search strategies can generally be categorized into two main types, namely black-box optimization techniques and one-shot techniques. Black-box methods operate independently of prior architectural knowledge and explore the entire search space to find optimal solutions, while one-shot techniques leverage prior knowledge to train new architectures, instead of training architectures from scratch. Studies involving black box methods have shown the ability to yield superior results. However, it is important to note that such methods often necessitate substantial investments of time and resources. In contrast, one-shot techniques may not achieve the same level of performance as black-box methods, nonetheless they are comparatively less resource intensive. This study incorporated a black-box search strategy named Bayesian optimization.

#### 2.2.3. Evaluation Strategy

The evaluation strategy plays a pivotal role in assessing the performance of diverse neural network architectures within the NAS process. Additionally, it is important to find an efficient evaluation strategy, as the computational expense associated with training and evaluating neural networks is generally higher. When deciding on an evaluation strategy, it is necessary to define an objective function or reward metric to quantify the performance of a given architecture, and the resulting performance metrics can be used as feedback to guide the NAS search strategy. This feedback mechanism informs the algorithm about process decision-making, either by facilitating the reinforcement of promising architectures or through the exploration of novel ones. The efficiency and efficacy of the NAS process are significantly influenced by the choice of evaluation strategy, encompassing factors such as the dataset employed, specific metrics measured, and frequency of model training and evaluation. Recent studies have demonstrated that performance estimation techniques, in contrast to traditional evaluation methods, offer enhanced efficiency within the NAS paradigm [28,29,30]. One prevalent approach involves leveraging a separate machine learning model to predict the performance of a given architecture based on its structural characteristics [8]. The evaluation strategy used in this study is elaborated in Section 3.5.3.

#### 2.2.4. Hardware-Aware Neural Architecture Search

The HW-NAS process defines a related set of constraints to the search space, the search strategy, and the evaluation criteria, to develop ANNs that strike an optimal balance between performance and resource consumption. It focuses on controlling the latency, memory utilization, and power consumption of the resulting ANN, while maintaining high accuracy [19]. This process incorporates domain knowledge on specific hardware platforms and their attributes, thereby refining the search space, search strategies, and the equilibrium between accuracy and hardware cost estimation within the NAS framework. Furthermore, it can be observed that the multifaceted nature of evaluation poses a challenge in identifying an optimal ANN architecture, as it necessitates the maximization of accuracy, while concurrently minimizing latency, memory consumption, energy consumption, and inference time, as well as the number of parameters and floating point operations (FLOPs).

### 2.3. Related Studies

There have been several studies conducted in the domain of ESC. Studies such as [16,23,26,31] used DL for ESC, while focusing on deploying DL-based audio classifiers on IoT devices. Out of all the studies related to DL-based ESC on resource-constrained edge devices, by Mohaimenuzzaman et al. [32], stands out as a state-of-the-art DL model that performed well under the aforementioned conditions.

Mohaimenuzzaman et al. [32] proposed ACDNet, a deep convolutional neural network architecture designed for raw audio classification. Unlike other state-of-the-art models that use pre-extracted features and a multi-channel input, ACDNet focuses solely on feature extraction through convolution layers. The network is fed with raw audio time series, i.e., single-channel input. ACDNet consists of two feature extraction blocks, followed by an output block. The feature extraction blocks are a spectral feature extraction block (SFEB) and temporal feature extraction block (TFEB). The SFEB extracts spectral features from the input audio signal, while the TFEB extracts temporal features. Both blocks consist of convolutional layers, followed by batch normalization and ReLU activation layers. The max-pooling layers have strides equal to their pool size, to avoid overlapping. The output block consists of a tiny dense layer followed by a softmax output layer. The tiny dense layer allows the network to adapt to changes in data shape when model compression is applied. The architecture is strongly motivated by the compressibility of the network and its adaptability for different audio lengths recorded at different sampling rates. Henceforth, this study adapts the search space and the building units of the proposed NAS process from this ACDNet architecture to search for a DL-based raw audio classification that would perform better than the state-of-the-art architectures.

Furthermore, the search strategy used in this study was influenced by the ColabNAS [33] process by Garavagno et al. This study presented an HW-NAS approach that can be used to produce lightweight task-specified CNNs. ColabNAS uses a cell-wise search space adapted from the VGG16 architecture, with a search strategy that explores this search space in two steps. In the first step, it adds cells until the generalization capability of the network increases according to Occam’s razor or until the hardware constraints are met. Next, it repeats the same process while changing the number of kernels, starting from a given number for better performance. The ColabNAS process was able to obtain state-of-the-art results for five image datasets from difficult domains. The novelty of this study is that we used the search strategy of ColabNAS for ESC, while changing the targeted input type from feature-extracted images to raw audio.

Moreover, several studies have been conducted exploring CNNs, TinyML, and NAS for audio classification tasks. One example was the study of NAS-based bird species recognition [34] by Muhling et al., where the authors proposed a novel NAS approach based on a memetic algorithm, to obtain a deep CNN that operates on the audio inputs. However, this study integrated a Gabor wavelet layer to extract the audio spectrograms. Moreover, in [12], Speckhard et al. proposed a NAS that optimized hardware considerations such as energy efficiency and memory usage, while maintaining the network accuracy. Their study proposed a search strategy utilizing both Bayesian and regularized evolutionary search, supplemented with early stopping techniques, while leveraging a random forest model for energy cost prediction. With this approach, the authors were able to construct CNNs with reduced resource consumption and enhanced accuracy compared to MobileNet-v2. Similarly, Lyu et al. [18] introduced an approach employing multi-objective NAS on resource-constrained edge devices, along with a novel search space improved from MobileNet-V2, which was scalable and practical for the selected target environment. Additionally, several studies have delved into the domain of hardware-aware NAS [19,35] in resource-constrained environments [36,37].

As is evident, building CNNs for raw audio classification for resource-constrained edge deployment and with state-of-the-art classification performance is a major challenge. Moreover, the current literature on NAS approaches for audio classification on resource-constrained edge devices is limited. In this study, we propose a novel HW-NAS process that results in a novel HW-NAS model with state-of-the-art performance, while addressing these limitations, and that is capable of providing a feasible solution for environmental sound classification deployed on an edge device. Furthermore, we prove that HW-NAS approaches can be successfully adapted to environmental sound classification by comparing the resulting models to existing popular edge-friendly CNNs.

## 3. Methodology

### 3.1. Process Flow

**Figure 1:**
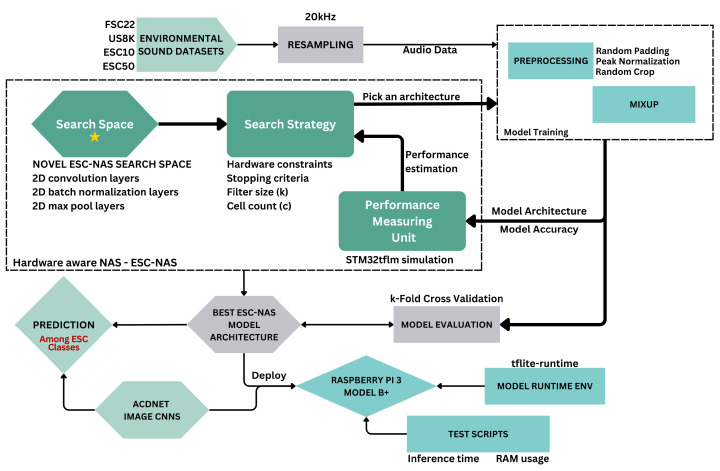
Workflow block diagram.

This study employed a HW-NAS process automating model architecture search to obtain a CNN architecture for raw audio classification on resource-constrained edge devices, thereby introducing a novel CNN architecture for environmental sound classification on edge devices. The experiments were conducted using publicly available popular environmental sound datasets, namely FSC22, UrbanSound8K, ESC-10, and ESC-50. This workflow comprised three primary subprocesses, namely the HW-NAS process referred to as ESC-NAS, the data augmentation and model training process, and the edge deployment process. A flowchart depicting the overall workflow of the experiment is shown in Figure 1.

Initially, the dataset selected from FSC22, UrbanSound8K, ESC-10, and ESC-50 was resampled to a fixed sample rate of 20 kHz. This fixed value was chosen as it contributed to the reduction in audio input size, model size, and resource consumption on edge devices. Moreover, no substantial performance degradation was observed when comparing the use of higher sampling rates for the audio input. The resampled dataset was subsequently employed in the model training process. This training process was integrated into the ESC-NAS procedure. Upon conclusion of the ESC-NAS process, the optimal model architecture identified by ESC-NAS was subjected to further training utilizing the same training process. The training process utilized the resampled dataset as input and generated training batches employing selected data processing techniques, including random padding, random cropping, and peak normalization, augmented with the mix-up technique. These training batches were dynamically constructed for each epoch within the training loop, ensuring adaptive and effective training iterations.

The novel contribution of this study lies in the NAS process. The ESC-NAS process is tasked with finding the best model architectures for raw audio classification CNNs, adhering to given resource constraints. The search strategy of ESC-NAS searches the specified novel search space to construct model architectures, which are subsequently trained using the designated training process. Following the training process, the trained model underwent performance evaluation via STM32tflm simulation. Based on the performance evaluation results and model accuracy metrics, the search strategy continued or stopped further exploration of model architectures. After the ESC-NAS process, it output the best ESC-NAS model architecture, characterized by optimal performance and compliance with predetermined environmental constraints.

The obtained best ESC-NAS model architecture was subsequently subjected to rigorous training using the designated training process, followed by evaluation through k-fold cross-validation. Therefore, the weights of the models trained during the ESC-NAS process were discarded and only their architectures were used without pretrained weights in the subsequent steps in the pipeline. Additionally, the predictive performance of the trained model was compared with seven selected models derived from the TinyML approach [38]. Furthermore, the trained model underwent deployment on the Raspberry Pi 3 B+ platform, where its RAM usage and inference time were tested.

### 3.2. Datasets

This study used four publicly available environment sound datasets. The utilized datasets and their characteristics were as follows.

The FSC22 dataset contains audio retrieved from FreeSound.org spanning 27 different classes. An ambiguity was identified during the manual inspection, yielding the removal of one class from the original dataset. The refined dataset consisted of 1950 audio clips related to forest environments distributed across 26 classes, with each class comprising 75 audio clips. Each audio clip is 5 s long and sampled at a 44.1 kHz sampling rate [39].

UrbanSound8K is another dataset that contains 8732 audio clips retrieved from FreeSound.org and that is distributed across 10 main classes related to urban environments, namely air conditioners, playing children, car horns, dog barks, engine handling, jackhammer, street music, siren, and gunshot. The duration of audio clips is 4 s or less, distributed unevenly across the classes [40].

The ESC-50 dataset contains 2000 audio clips, which are equally distributed across 50 classes. The 50 classes are subdivided into five major sections namely, animal sounds, urban noises, natural soundscapes and water sounds, human (non-speech) sounds, and domestic sounds. Each audio clip is 5 s long and is sampled at a 44.1 kHz sampling rate [41].

The ESC-10 dataset is a subset of the ESC-50 dataset. It contains 10 classes, namely chainsaw, clock tick, cracking fire, crying baby, dog, helicopter, rain, rooster, sea waves, and sneezing. As in ESC-50, each audio clip is 5 s long and sampled at a 44.1 kHz sampling rate.

### 3.3. Data Preprocessing

Data preprocessing was conducted to standardize the audio data across all datasets involved in this study. First, the audio clips were resampled at a fixed sampling rate of 20 kHz, aiming to reduce the input size, model size, and FLOPs. Following that, the resampled data were subjected to standardization by making the duration of the audio clips 5 s. This ensured equal importance when applying data preprocessing techniques such as random padding and random cropping. In the case of an audio clip with a duration less than 5 s, the audio clip was repeated until a duration of 5 s was achieved. Additionally, amplitude normalization was applied following Equation (1) based on a 16-bit float representation of the audio signal. In Equation (1), *S* is the input audio array representation, while $S_{n}$ is the normalized audio array representation.

(1) $$S_{n}=\frac{S}{2^{16-1}}$$

### 3.4. Data Augmentation

We used data augmentation to increase the number of training data records. Mix-up is a data augmentation technique that can synthetically increase the size and diversity of an acoustic dataset. This study used mix-up to dynamically augment the dataset when creating training batches for each epoch during the training process. In a related work by Mohaimenuzzaman et al. [32], mix-up was referred to as the mixing up of randomly selected audio clips from disparate classes in a randomized ratio to generate a synthesized audio clip. The synthesized audio clip, $S_{mix}$ can be obtained using Equation (3) as of [42], where $S_{1}$ and $S_{2}$ are two randomly selected audio clips from two distinct classes whose maximum gains are $g_{1}$ and $g_{2}$, respectively. In Equation (2), *r* refers to a random value between 0 and 1.

(2) $$p=\frac{1}{1+10*\frac{g_{1}-g_{2}}{20}*\frac{1-r}{r}}$$

(3) $$S_{mix}=\frac{pS_{1}+\left(1-p\right)S_{2}}{\sqrt{p^{2}+{\left(1-p\right)}^{2}}}$$

Incorporating mix-up into the ESC-NAS pipeline entailed a distinctive aspect characterized by the dynamic construction of training batches during each epoch within the training loop. This process involved randomly selecting data points from the original training dataset and utilizing mix-up to generate augmented training data points, thereby enhancing the diversity of the training set.

### 3.5. Hardware-Aware Neural Architecture Search

This study proposed a novel HW-NAS framework named the ESC-NAS process, which can be used to tailor task-specific and lightweight CNNs. ESC-NAS features a unique search space, optimization problem formulation, and search strategy. Inspired by ACDNet [32], ESC-NAS employs a cell-based search space and a search strategy based on Colab NAS [33], employed using simulation-based hardware cost evaluation. Notably, ESC-NAS specializes in designing raw audio classification models, wherein feature extraction is exclusively conducted through convolutional layers, as inspired by ACDNet. This approach optimizes the resulting models for edge applications, enabling the accommodation of raw audio input, without the resource-intensive application of feature extraction techniques.

#### 3.5.1. Search Space

**Figure 2:**
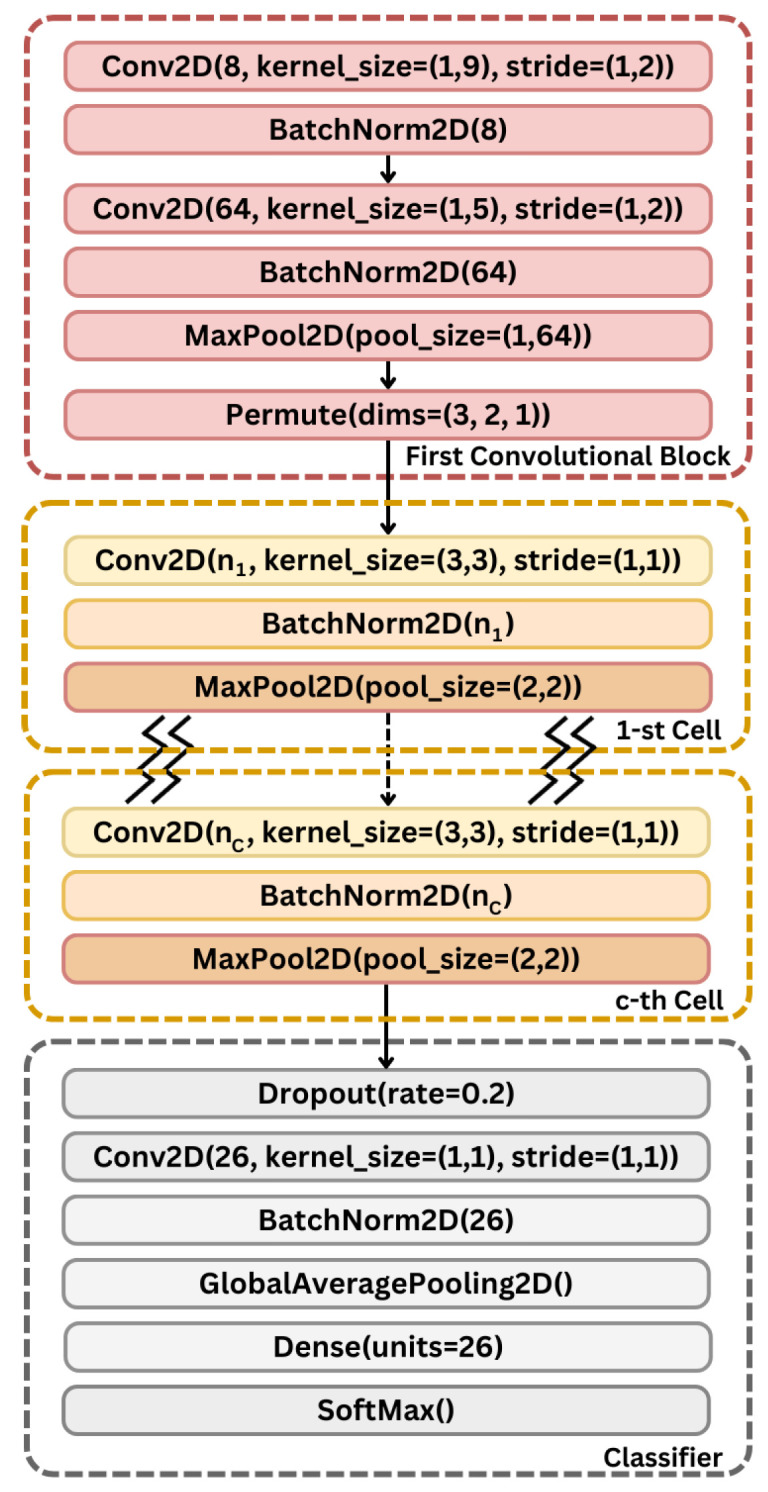
Search space of ESC-NAS.

ESC-NAS uses a cell-based search space to construct the heart of the CNN model depicted in yellow in Figure 2, based on the output of the preceding layers. The search space was based on the TFEB module of [32], as it was proven to extract distinctive features from audio data that aid in their classification. These repeating cells convolve over both time and frequency, similarly to a convolution on images, where one repeating cell consists of a 2D convolution layer, a 2D batch normalization layer, a rectified linear (ReLU) activation layer, and a 2D max pooling layer. The 2D convolution layer has k filters determined by the search strategy, a kernel size of (3,3), and a stride of (1,1), and it is 1 padded. This layer is responsible for extracting the temporal features from the input. The batch normalization layer reduces overfitting and enhances the generalizability of the model. A ReLU activation layer was added to introduce non-linearity to the model. Finally, the 2D max pooling layer is used to extract the most important features from the input, while reducing the dimensions of the features, thereby reducing the computational complexity. In contrast to the TFEB module in [32], which used a similar architecture to VGG-13, the proposed search space cell has only one 2D convolution layer, 2D batch normalization layer, and ReLU activation layer, followed by one 2D max pooling layer. This eliminates the addition of extra parameters in recurrent convolutional layers with identical filter counts. This process is overseen by the search strategy, which increments the number of repeating cells, granting us precise control over how these cells are combined during model construction.

#### 3.5.2. Search Strategy

**Figure 3:**
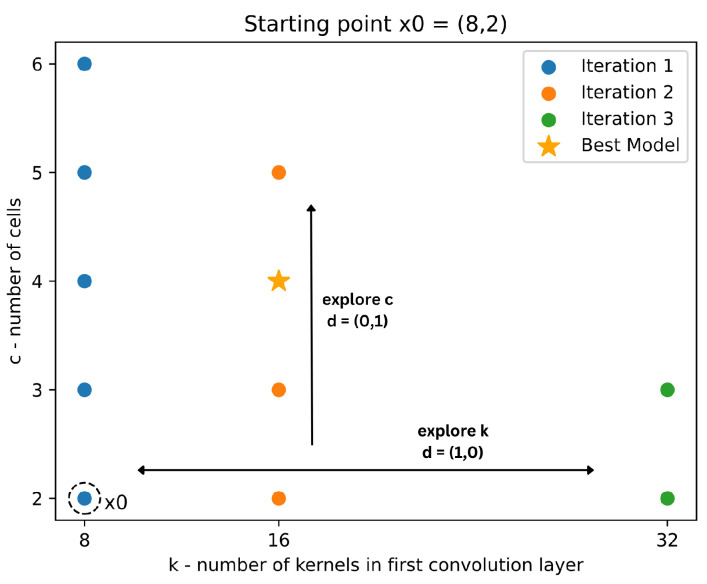
Search strategy of ESC-NAS.

The ESC-NAS process uses Bayesian optimization, a black-box search strategy that modulates the growth of the cell count and the filter count to find the best possible model architecture from the search space, while satisfying the hardware constraints. Formulated as a constrained optimization problem, ESC-NAS prioritizes maximizing the validation accuracy, while satisfying peak RAM and flash memory constraints that are dependent on the cell count, filter count, and other model parameters. Initially, the search strategy explores different configurations of repeating cells for a given base filter count, incrementally adjusting until a satisfactory model performance or hardware constraint is reached. Subsequently, the process repeats with varying base filter counts in the convolutional layers. This process is illustrated in Figure 3, where *k* denotes the base filter count of the convolution layers in the repeating cells and *c* denotes the number of repeating cells. Subsequently, the resulting model architecture with a base filter count of *k* has 2∗k filters in the convolution layer in the first repetitive cell block, followed by cell blocks with an increased filter count by a factor of *k*. The ESC-NAS process starts with the initial parameter values of k=8 and c=2, selected based on manual examination of ESC-NAS process behaviors, to expedite convergence towards the optimal model architecture. With these initial values, the lower bounds for *k* and *c* are set to 1 and 2, respectively. The upper bounds for *k* and *c* are determined by the hardware constraints imposed by the user, based on the specific hardware configuration on which the resulting models are intended to be deployed. Furthermore, Occam’s razor is used as the stopping criterion, to prohibit excessive growth of the model complexity. The search strategy employed was derived from Garavagno et al. [33], with comprehensive mathematical modeling and the problem formulation elaborated in their work.

#### 3.5.3. Evaluation Strategy

The evaluation criteria of ESC-NAS encompassed the validation accuracy of the model, peak RAM usage, and flash memory consumption, to obtain the optimal CNN architecture. A hardware simulation was employed to estimate the hardware and computational costs of the obtained model and to verify compliance with the specified hardware constraints. Consequently, the computed metrics estimating hardware and computational costs are fed back to the search strategy to select a model with an optimal trade-off between model performance, complexity, and resource consumption. This approach is more accurate compared to ML-based estimation techniques, since it closely resembles real-world implementations, offering more nuanced insights into the operational dynamics of the resulting CNN on an edge device. Specifically, the X-CUBE-AI software [43], from ST Microcontrollers was utilized for simulating the performance of the CNNs derived from ESC-NAS on a highly resource-constrained ST Microcontroller-based platform. This provided an accurate estimation of the peak memory consumption and the flash occupancy of the model in a highly resource-contained ST microcontroller-based platform. As the experimental setup of this study used a Raspberry Pi 3 Model B+, the peak RAM and flash memory constraints were relaxed to 5 MB and 150 kBs.

#### 3.5.4. Resulting Architecture

The ESC-NAS process yielded the optimal model architecture capable of achieving superior performance, while maintaining compliance wit the specified hardware constraints. A generic representation of the resulting architecture is illustrated in Figure 2. The initial feature extraction block, which is depicted inside the red dotted box in Figure 2, was adapted from the spatial feature extraction block (SFEB) in ACDNet [32]. This contains two convolution layers with 8 and 64 filters, respectively, with 1D kernels and strides. These convolution layers are followed by a batch normalization layer, a ReLU activation layer, and a 2D max pooling layer. Consequently, a permutation layer is used to swap the axis in the output from the max pooling layer, such that the succeeding layers convolve over both time and frequency dimensions. Subsequently, the output of the permutation layer is fed into the repeating blocks of cells added from the ESC-NAS search space. The fundamental cell structure of the ESC-NAS search space was based on [32], where the core component of a cell is the 2D convolution layer, which is followed by a batch normalization layer and a 2D max pooling layer.

Lastly, the classifier block contains a dropout layer to reduce overfitting, a 2D convolution layer with a filter count equal to the number of classes in the classification, a batch normalization layer, a global average pooling layer, a fully connected layer with units equal to the number of classes, and finally softmax activation. This block is similar to the final layers of the TFEB from [32], and in Figure 2, which shows the generic architecture, this is highlighted in grey color, and is similar to the final layers in TFEB in ACDNet [32].

**Figure 4:**
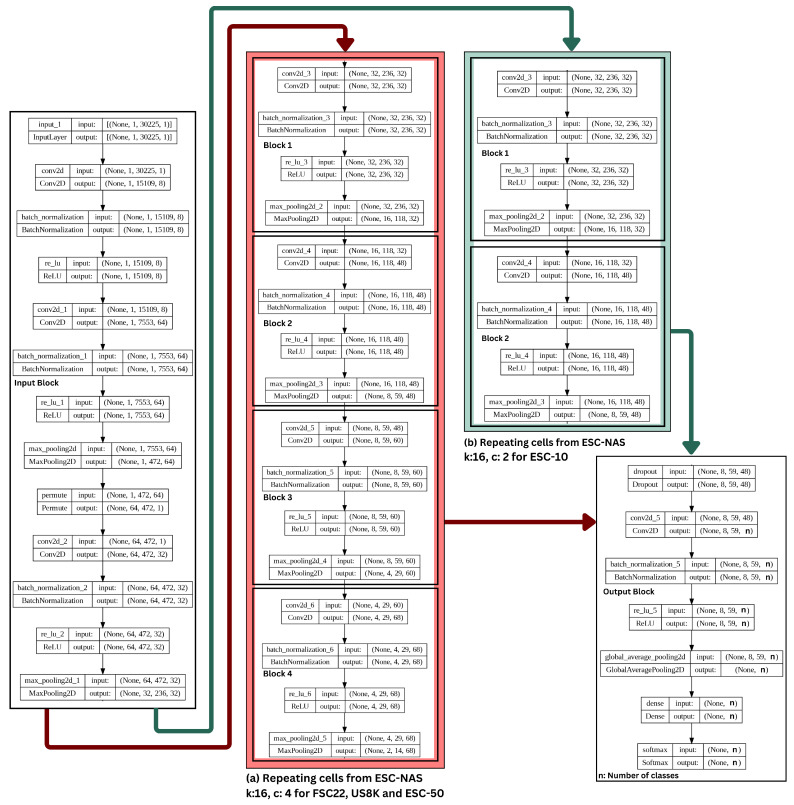
Resulting architectures from ESC-NAS for different datasets. (**a**) k: 16, c: 4 for FSC22, US8K, and ESC-50 (**b**) k: 16, c: 2 ESC-10.

In this experiment, the ESC-NAS process conducted on the selected FSC22, US8K, and ESC-50 datasets resulted in the architecture shown in Figure 4a, with a base filter count of 16 (k=16) and a cell count of 4 (c=4), as the architecture which gave the maximum validation accuracy, while adhering to the hardware constraints. From the ESC-NAS process conducted on the ESC-10 dataset, the resulting best model architecture depicted in Figure 4b was characterized by a base filter count of 16 (k=16) and a cell count of 2 (c=2), where *n* denotes the number of classes in the dataset. In Figure 4, the input and output blocks for the two model architectures are common; however, the repeating cell count in the central block is different.

Following the ESC-NAS process, the obtained best model architectures for the FSC22, ESC-10, and ESC-50 datasets underwent training with 5-fold cross-validation, while the best model architecture for US8K was subjected to training with 10-fold cross-validation.

### 3.6. Implementation Details

The code implementation of this study involved utilizing functions provided by the Librosa [44] and Audiomentations [45] libraries for audio preprocessing. The ESC-NAS process was implemented using Python libraries such as Numpy [46] and the TensorFlow framework [47]. The final model training was performed using Pytorch [48] and the models were converted to TensorFlow Lite format to be deployed on edge devices.

**Figure 5:**
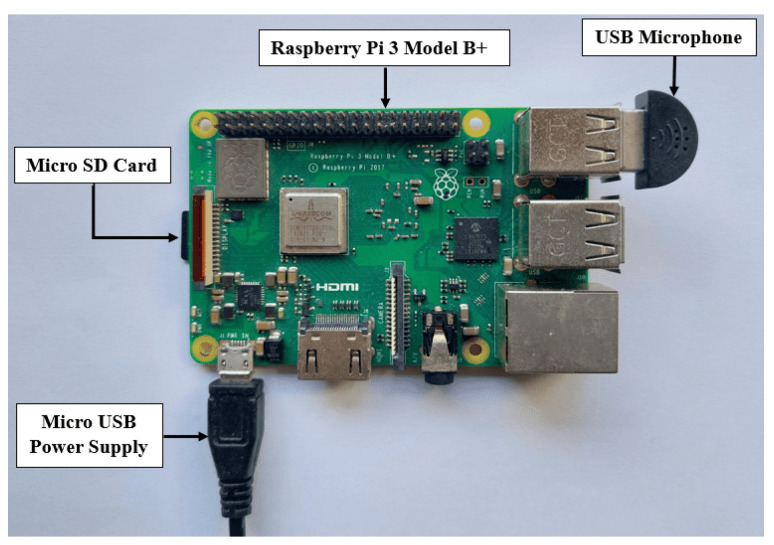
Device setup.

In the experiment, the trained models obtained from the ESC-NAS process were evaluated on an edge device to ascertain the performance of the model architectures within their targeted deployment environments. The experimental setup was constructed using a Raspberry Pi 3 Model B+ with the 32-bit legacy Raspberry Pi OS, as depicted in Figure 5. The operating system was burned to a 16 GB class 10 micro SD card, and the required dependencies and libraries were installed in a Python virtual environment. Additionally, a 12.5 W micro-USB power supply with a 2.5 A output rating was utilized to power the device, ensuring the optimal performance of both the device and its components.

The models obtained were deployed on the edge device as TensorFlow Lite models, utilizing the tflite-runtime. Subsequently, these deployed models underwent rigorous testing to assess the inference time per audio clip, memory consumption for inference, and accuracy of the on-device predictions. To ensure the reliability of these metrics and mitigate the influence of biases and outliers, multiple experiments were conducted. The average values of these metrics were then calculated based on the results obtained from the test scripts executed on the trained best models.

## 4. Results and Analysis

This section evaluated the ESC-NAS process on popular environmental sound classification datasets. The ESC-NAS was executed for each of these datasets separately, and the resulting best model architecture was selected for training using mix-up augmentation.

During the experiments conducted with the proposed ESC-NAS process across multiple datasets, consistent observations were made, irrespective of the dataset used. Notably, the memory consumption of the trained model architectures resulting from the ESC-NAS process on the simulated microcontroller was found to be primarily influenced by the base filter count (*k*). As the value of (*k*) increased, there was a corresponding decrease in the memory required to store tensors, resulting in a reduced estimated memory consumption. This memory consumption trend was consistently evident across all iterations. Additionally, it was observed that the flash memory required to store the model architectures increased with higher block counts (*c*) and base filter counts (*k*), aligning with expectations. However, there was no discernible pattern for the accuracy during the iterations of the ESC-NAS process for each dataset, indicating a dependency on the complexity of the dataset. Moreover, it is noteworthy that the accuracies reported after each iteration for all datasets were below average. This was due to the limitation imposed on the training epochs within the ESC-NAS process. To enhance the efficiency of the ESC-NAS process, the training epochs were restricted to 200. However, the optimal performance could be obtained by training the best model identified from the ESC-NAS iterations for about 2000 epochs [32].

### 4.1. Results of FSC22 Dataset

With the FSC22 dataset, it was observed that the ESC-NAS process evaluated 11 model architectures, starting with a base filter count of 8 and a cell count of 2, as shown in Table 1.

From Table 1, it is evident that increasing the values of *k* and *c* correlated with improved model accuracy. Consequently, model architectures with a base filter count smaller than the base value of 8 were not explored. However, the augmentation of *k* and *c* was constrained by the imposed resource limitations. This constraint was evident in iterations 8 and 10, where the flash memory constraint was violated, leading to a halt in the increment of the cell count and the resulting model architectures remaining untrained, prompting the ESC-NAS search strategy to reassess the base filter count with a cell count of 2 in the subsequent iteration. Although the accuracy of the models was improved with the increasing cell count and the obtained models were within the desired hardware constraints, the cell count could not be increased continuously. In iteration 4, for instance, a model with a cell count of 6 was not evaluated, due to the output feature dimensions from the final 2D convolution layer in the last cell becoming less than or equal to 1, thus preventing further convolution in the subsequent output blocks. Moreover, the base filter count was not escalated beyond 32, due to the infeasibility of a model architecture with a base filter count of 32 and a cell count of 3, imposed by the resource constraints. Additionally, it can be noted that a higher number of repetitive cells was associated with an improved model performance. Ultimately, the ESC-NAS process discovered the best model architecture for the FSC22 dataset in the 7th iteration, with an optimal trade-off between accuracy and architectural complexity, and with a base filter count of 16 and a cell count of 4.

### 4.2. Results of UrbanSound8K Dataset

The ESC-NAS process evaluated 11 model architectures, starting with a base filter count of 8 and a cell count of 2, as shown in Table 2 for the UrbanSound8K dataset.

Table 2 demonstrates that increasing the base filter count and repetitive cell count was associated with an enhanced model accuracy and increased resource consumption. Therefore, model architectures with a base filter count smaller than 8 were not investigated further. In iteration 4, the model with a cell count of 6 was not evaluated, because the output feature dimensions from the final 2D convolution layer in the 6th cell became less than or equal to 1, preventing further convolution in the subsequent output blocks. In iterations 8 and 10, the increment of cell count was halted with the violation of the flash memory constraint in the hardware simulation. Subsequently, the ESC-NAS search strategy shifted to evaluating different base filter counts, commencing with a cell count of 2 in the subsequent iteration. However, the base filter count of 64 was not explored in the ESC-NAS process, due to infeasibility arising from resource constraints. Lastly, the ESC-NAS process achieved the optimal model architecture for the UrbanSound8K dataset in the 7th iteration, with an optimal trade-off between accuracy and architectural complexity, and with a base filter count of 16 and a cell count of 4.

### 4.3. Results of ESC-10 Dataset

For the ESC-10 dataset, it could be observed that the ESC-NAS process evaluated 7 model architectures, starting with a base filter count of 8 and a cell count of 2, as shown in Table 3.

It is evident from Table 3, that the increase in the base filter count did not uniformly correspond to an improved model accuracy when the count was set to 8. Moreover, a notable decrease in accuracy was also observed with this dataset, when the cell count was increased from 3 to 4 during the second iteration, which caused the discontinuation of further exploration for a filter count of 8. Subsequently, a similar trend was observed for a base filter count of 16. However, when comparing the performance of models with equal cell counts of base filters of 8 and 16, it can be observed that the increase in the filter count led to an increase in model performance. Therefore, model architectures with a smaller base filter count than 8 were not explored. Consequently, in iteration 6, violation of the flash memory constraint halted the cell count increment. The ESC-NAS process concluded there, as the model performance increased with the filter count. Finally, the ESC-NAS process achieved the optimal model architecture for the ESC-10 dataset, with an optimal trade-off between accuracy and architectural complexity, in the 3rd iteration, with a base filter count of 16 and a cell count of 2.

### 4.4. Results of ESC-50 Dataset

The ESC-NAS process assessed 7 model architectures, starting with a base filter count of 8 and a cell count of 2, as outlined in Table 4 focusing on the ESC-50 dataset.

As depicted in Table 4, incremental adjustments to both the base filter count *k* and cell count *c* exhibited a positive correlation with enhanced model accuracy across the iterations. Consequently, models with filter counts smaller than the base count of 8 were not explored, reflecting a strategic focus on model architectures with potentially higher predictive capabilities. However, the augmentation of *k* and *c* was governed by hardware constraints, as demonstrated in iterations 8 and 10, where the flash memory constraint impeded further increases in cell count. Moreover, untrained model architectures resulted, prompting the ESC-NAS strategy to reassess the base filter count, initiating subsequent iterations with a cell count of 2. Although the increment in the cell count while adhering to the resource constraints improved the model performance, there was a limit to the feasibility of the model architecture. In iteration 4, a mode architecture with a cell count of 6 was precluded from evaluation, due to diminished output feature dimensions, hindering further convolution in the final output block. Furthermore, the base filter count was capped at 32, as a model configuration with a base filter count of 32 and a cell count of 3 proved unviable within the resource constraints. Lastly, it can be noted that a higher prevalence of repetitive cells was associated with improved model performance. Ultimately, the ESC-NAS process obtained the optimal model architecture for the ESC-50 dataset at the 7th iteration, with an optimal trade-off between accuracy and architectural complexity, characterized by a base filter count of 16 and a cell count of 4.

Table 5 shows the statistics of the resulting models after training the best model architectures for each dataset. The training process was conducted with mix-up augmentation for 2000 epochs, and k-fold cross-validation was used to evaluate the model performance across the whole dataset. The mean validation accuracy was obtained by training and evaluating the models across all folds of each dataset, called the cross-validation (CV) score. It can be observed that the model architectures with a base filter count of 16 and a cell count of 4 performed best for the FSC22, UrbanSound8K, and ESC-50 datasets, while a model architecture with a base filter count of 16 and a cell count of 2 was sufficient to classify the ESC-10 dataset with a comparatively higher CV score. This discrepancy arose from the inherent complexity of the FSC22, UrbanSound8K, and ESC-50 datasets, characterized by a larger number of classes compared with ESC-10, which demands model architectures with greater complexity and capacity for effective classification. Conversely, for the ESC-10 dataset, which represents a less intricate classification task, a simpler architecture sufficed to achieve satisfactory performance. Moreover, it can be noted that the models obtained for the FSC22, UrbanSound8K, and ESC-50 datasets had a similar architectural complexity, although there were minor differences present, due to the number of audio classes and the nature of the audio in the different datasets.

Consequently, the ESC-NAS model obtained for the FSC22 dataset was deployed on the edge device setup described in Section 3.6. Since this edge deployment aimed to prove the compatibility of ESC-NAS model architectures on edge devices, the aforementioned model was selected among the similarly characterized models obtained from US8K and ESC-50.

The proposed novel search space of ESC-NAS process is based on the ACDNet [32] model architecture. Therefore, a comparison was conducted with the compressed ACDNet, Micro-ACDNet, trained on the FSC22 and ESC-NAS model obtained for FSC22. This comparison aimed to monitor the performance of the two models on a Raspberry Pi 3B+, as well as on a personal computer with 11th Gen Intel^(R)^ Core^(TM)^ i7-1165G7 @ 2.80 GHz, 16.0 GB RAM and 512 GB SSD. Table 6 shows the performance of the selected models in both targeted deployment environments, focusing on the peak memory (RAM) usage and inference time.

**Figure 6:**
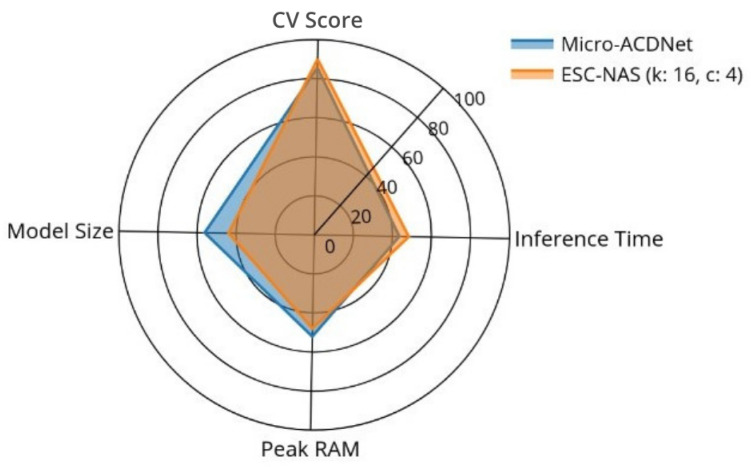
Comparison of the performance metrics of the compressed ACDNet and ESC-NAS models for the FSC22 dataset.

Moreover, Table 6 shows that the peak memory consumption of the models on both the PC and the Raspberry Pi 3 B+ remained nearly consistent, regardless of the model architecture, parameter count, and FLOPs. However, the inference time on the Raspberry Pi was notably higher than on the PC, due to the comparatively limited computational capabilities of the Raspberry Pi 3 B+. The ESC-NAS model demonstrated better peak memory usage compared with the Micro-ACDNet model on both devices. Although the inference time of the ESC-NAS model slightly surpassed that of the Micro-ACDNet model, this can be considered insignificant in practical applications such as environmental observatories and urban monitoring. Additionally, Figure 6 illustrates that the model size of Micro-ACDNet was larger than that of ESC-NAS, while conversely, the accuracy of Micro-ACDNet was inferior to that of ESC-NAS. Therefore, it can be concluded that the ESC-NAS model achieved a better trade-off between model performance and complexity compared to the current state-of-the-art raw audio classifier for edge devices, thus proving the effectiveness of the ESC-NAS for raw audio classification on edge devices.

## 5. Discussion

### 5.1. Study Contribution

This study proposed a novel HW-NAS named ESC-NAS, which can be employed to design and develop DL-based audio classification models for raw audio classification on resource-constrained edge devices. Moreover, the study proposed a novel model architecture through the ESC-NAS process for time- and resource-efficient audio classification on edge devices.

The importance of the proposed ESC-NAS process lies in its capability to discover lightweight task-oriented deep CNN model architectures. The notable speciality of the resultant model architectures is that these models do not necessitate the utilization of any feature engineering techniques, as the models can solely rely on the convolution layers for feature extraction. Furthermore, the obtained models exhibited optimal performance, alongside reduced resource consumption, making these models suitable for edge deployment.

Additionally, the ESC-NAS process introduced a novel cell-based search space consisting of 2D convolution, 2D batch normalization, and 2D max pooling. The search space demonstrated exceptional feature extraction capabilities by utilizing minimal filters and cell counts, as evidenced by the high performance across various environmental sound datasets, as illustrated in Table 5. The search strategy explores this search space, intending to find model architectures with an optimum trade-off between model performance and resource consumption. Based on the iterations conducted with ESC-NAS across diverse datasets, it was observed that higher filter counts and cell counts tended to yield models with better performance. However, the growth in model complexity was regulated by the hardware cost simulation. This facilitated the search strategy in identifying model architectures that struck an optimal balance between accuracy and resource utilization. Moreover, the Occam’s-razor-based stopping criteria associated with the search strategy influenced the effective convergence towards the best model architectures, thereby improving the efficiency of the overall NAS process.

Consequently, the resultant model architectures underwent thorough training with k-fold cross-validation, after which they were deployed on resource-constrained edge devices to validate their performance in the intended deployment environments. In order to verify that the performance of the model architectures obtained from the ESC-NAS process was suitable for edge deployment, the ESC-NAS model obtained for FSC22 was deployed on a Raspberry Pi 3 B+ and compared against the Micro-ACDNet architecture trained on the same dataset. The comparative analysis, outlined in Table 6, underscored the viability of ESC-NAS for constructing high-performing and less resource-consuming DL architectures for environmental sound classification using raw audio on resource-constrained edge devices.

### 5.2. Comparison with Existing Studies

Existing studies involving environmental sound classification using raw audio are limited. One of the key studies that designed an environmental sound classification model for raw audio was by Mohaimenuzzaman et al. [32]. This study introduced a comprehensive pipeline to compress DL-based audio classification models for resource-constrained edge devices. The pipeline employs techniques such as pruning and quantization to achieve compression. Two resultant models from this pipeline are Micro-ACDNet, a compressed version of the ACDNet model architecture proposed by Mohaimenuzzaman et al., and Micro-AclNet, a compressed version of the AclNet introduced by Huang et al. [49], as an efficient end-to-end raw audio classification model. Moreover, Ranasignhe et al. [17], proposed an HW-NAS process tailored for audio classification in resource-constrained environments. A notable characteristic of the HW-NAS models is their general inapplicability for raw audio classification, as they require feature-extracted audio data arrays as input. Here, we compared the novel ESC-NAS model architectures with the aforementioned three models, namely Micro-ACDNet, Micro-AclNet, and HW-NAS.

When the ESC-NAS model obtained for FSC22 was compared with Micro-ACDNet trained for FSC22 and HW-NAS [17] model for FSC22, it was observed that the ESC-NAS model had the highest CV score. As shown in Table 7, which compares the model size, parameter count, and CV scores of these models, the ESC-NAS model with a base filter count of 16 and 4 repetitive cells achieved a higher cross-validation score of 85.78% compared to the 85.64% of Micro-ACDNet and 83.05% of HW-NAS. While the resulting ESC-NAS model exhibited a lower complexity compared to the Micro-ACDNet model, it is worth noting that the HW-NAS model demonstrated a lower parameter count and a model size comparable to that of ESC-NAS. However, the HW-NAS model lacked the capability for raw audio classification and required significant computational resources from the edge device for feature extraction [17]. Consequently, the ESC-NAS model was the most suitable for audio classification on resource-constrained edge devices, offering a better performance with lower resource consumption.

Furthermore, a comparative analysis between the ESC-NAS models acquired from various datasets and Micro-ACDNet and Micro-AclNet [32] models trained on corresponding datasets revealed that ESC-NAS exhibited comparable or superior performance across the diverse datasets. As illustrated in Table 8, the ESC-NAS models demonstrated the highest cross-validation scores for the FSC22 and UrbanSound8K datasets, comparable performance to Micro-ACDNet for ESC-10, and a slight decrease in performance of 2.65% compared to Micro-ACDNet for ESC-50.

**Figure 7:**
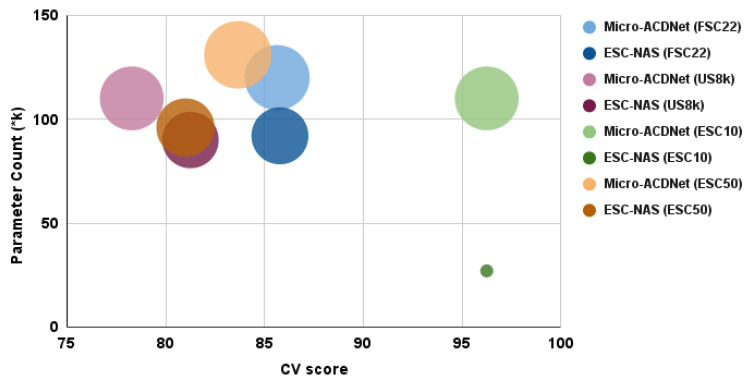
Comparison of CV score, parameter count, and model size of compressed ACDNet and ESC-NAS models for different datasets. The circle radius represents the model size.

However, upon examination of Figure 7, depicting model CV score plotted against parameter count in thousands (*k) and model size, which is denoted by circle radius for both ESC-NAS and Micro-ACDNet models, it becomes apparent that the ESC-NAS models offered the most favorable trade-off between model performance and complexity. In Figure 7, the Micro-ACDNet and the ESC-NAS models related to the same dataset are represented by varying shades of the same base color.

Thus, considering the four environmental sound classification datasets examined in this study, the ESC-NAS models demonstrated comparable accuracies to existing approaches, while exhibiting significantly reduced complexity. Moreover, the ESC-NAS models offered an optimal balance between model performance and complexity for raw environmental sound classification on resource-constrained edge devices, surpassing state-of-the-art models, as illustrated in Figure 7. Notably, ESC-NAS models are well-suited for deployment on edge devices like the Raspberry Pi 3 B+ and other highly resource-constrained platforms such as microcontrollers, due to their minimal peak RAM usage and compact model size. Furthermore, by eliminating complex feature extraction procedures and spectrogram generation, the ESC-NAS models achieved reduced resource consumption and lower latencies compared to prior work employing models originally designed for image classification [38]. In summary, ESC-NAS models, specifically tailored for environmental sound classification on resource-constrained edge devices, present a promising solution, characterized by high performance and resource efficiency.

### 5.3. Challenges and Future Work

This study aimed to develop a CNN-based audio classification architecture optimized for edge deployment through a hardware-aware NAS process. In the experiments conducted using the device setup outlined in Figure 5, we observed that noise and distortion significantly impacted the quality of audio recordings captured by the microphone. However, high-quality microphones capable of mitigating these issues typically exhibit a high power consumption and resource utilization, posing a considerable challenge to their practical deployment for real-time audio classification, despite the robust classification capabilities of the underlying models. Moreover, in resource-constrained and harsh environments like forests, where communication infrastructure is limited, ensuring reliable communication between edge devices and external entities becomes a major concern. Recent advancements in IoT and communication technologies, such as LoRA, offer feasible solutions to address these challenges [50].

Accordingly, these challenges can be addressed by employing mitigation approaches such as real-time monitoring of the edge devices with technologies such as GPS and regular status inquiries, using more robust hardware with protective enclosures, implementing cybersecurity measures such as encryption and access monitoring, and ensuring redundancy in critical components of the system. The proposed ESC-NAS process demonstrated adaptability to varying application contexts by leveraging domain-specific datasets tailored to each application domain. In addition, it has flexibility in adjustment of model parameters and resource constraints. Moreover, in real-world applications, the protection of these edge devices from theft, natural hazards, and malicious attacks is a major challenge in forest observatory applications. For real-time monitoring using technologies such as GPS and regular status inquiries, robust hardware with protective enclosures and the implementation of cybersecurity measures, including encryption and access monitoring, can be used to mitigate these challenges. Furthermore, it is vital to ensure redundancy in critical components, to enhance the resilience of such observatory systems.

## 6. Conclusions

This study presented a novel hardware-aware neural architecture search process to design deep learning models to classify raw audio with the optimal trade-off between model performance and complexity. The proposed novel search space, inspired by ACDNet, is highly capable of extracting features from raw audio using a simple cell-based structure. It obtained state-of-the-art classification performance on diverse environmental sound datasets, surpassing the CNN compression solutions, while having a lower resource consumption. The highest performance was obtained for the FSC22, UrbanSound8K, and ESC-50 datasets with a model with a base filter count of 16 and a cell count of 4, while an ESC-NAS model with a cell count of 2 was sufficient to classify the ESC-10 dataset. The ESC-NAS model achieved cross-validation scores of 85.78%, 81.25%, and 81.0% for the FSC22, UrbanSound8K, and ESC-50 datasets, respectively, with a model size of around 365 kBs, while for the ESC-10 dataset, the ESC-NAS model archived a cross-validation score of 96.25%, with a model size of just 111 kBs.

ESC-NAS also provides task-specific lightweight CNNs that consumed 0.5 MB less peak RAM than comparable micro-ACDNet models obtained by CNN compression for the FSC22 dataset. Compared to CNNs that use spectrograms to classify audio such as MobileNetV3, ESC-NAS models are highly resource-efficient due to the removal of explicit feature extraction. Thereby proving the feasibility of utilizing ESC-NAS for environmental sound classification using audio, for designing lightweight custom raw audio classifiers targeting resource-constrained edge devices.

## Figures and Tables

**Table 1:** ESC-NAS iteration results for FSC22.

Model Step	Base Filer Count	Block Count	Estimated RAM Usage (MB)	Estimated Flash Memory (kB)	Accuracy
0	8	2	2.31	23.21	44.36%
1	8	3	2.31	31.35	49.49%
2	8	4	2.31	42.16	62.82%
3	8	5	2.31	55.05	64.1%
4	8	6	2.31	77.58	-
5	16	2	1.16	39.71	53.33%
6	16	3	1.16	67.72	60.51%
**7**	**16**	**4**	**1.16**	**106.39**	**68.21%**
8	16	5	1.16	152.89	-
9	32	2	0.58	92.63	60.0%
10	32	3	0.58	198.53	-

The row with the bold font shows the best iteration, which has the highest accuracy.

**Table 2:** ESC-NAS iteration results for UrbanSound8K.

Model Step	Base Filer Count	Block Count	Estimated RAM Usage (MB)	Estimated Flash Memory (kB)	Accuracy
0	8	2	2.31	21.77	38.37%
1	8	3	2.31	29.87	44.33%
2	8	4	2.31	40.38	46.39%
3	8	5	2.31	53.42	52.35%
4	8	6	2.31	75.45	-
5	16	2	1.16	37.74	44.1%
6	16	3	1.16	65.7	55.44%
**7**	**16**	**4**	**1.16**	**104.26**	**62.66%**
8	16	5	1.16	150.73	-
9	32	2	0.58	89.9	49.48%
10	32	3	0.58	195.55	-

The row with the bold font shows the best iteration, which has the highest accuracy.

**Table 3:** ESC-NAS iteration results for ESC-10.

Model Step	Base Filer Count	Block Count	Estimated RAM Usage (MB)	Estimated Flash Memory (kB)	Accuracy
0	8	2	2.31	21.97	15.0%
1	8	3	2.31	29.93	37.5%
2	8	4	2.31	40.61	25.0%
**3**	**16**	**2**	**1.16**	**37.78**	**60.0%**
4	16	3	1.16	65.70	31.25%
5	32	2	0.58	90.10	20.0%
6	32	3	0.58	195.62	-

The row with the bold font shows the best iteration, which has the highest accuracy.

**Table 4:** ESC-NAS iteration results for ESC-50.

Model Step	Base Filer Count	Block Count	Estimated RAM Usage (MB)	Estimated Flash Memory (kB)	Accuracy
0	8	2	2.31	26.45	37.0%
1	8	3	2.31	34.65	51.0%
2	8	4	2.31	45.48	55.5%
3	8	5	2.31	58.45	61.5%
4	8	6	2.31	58.48	-
5	16	2	1.16	43.21	49.5%
6	16	3	1.16	71.59	58.25%
**7**	**16**	**4**	**1.16**	**110.52**	**65.5%**
8	16	5	1.16	157.13	-
9	32	2	0.58	97.4	55.75%
10	32	3	0.58	203.85	-

The row with the bold font shows the best iteration, which has the highest accuracy.

**Table 5:** ESC-NAS best models for different environmental sound datasets.

Dataset	Model Architecture	Statistics	
FSC22	k:16, c:4	CV Score	85.78 ± 0.39%
		Parameters	92,422
		Model Size	365 kB
UrbanSound8K	k:16, c:4	CV Score	81.25 ± 2.47%
		Parameters	90,678
		Model Size	359 kB
ESC-10	k:16, c:2	CV Score	96.25 ± 0.22%
		Parameters	27,326
		Model Size	111 kB
ESC-50	k:16, c:4	CV Score	81.00 ± 1.35%
		Parameters	95,998
		Model Size	379 kB

k: Base filter count of the repetitive cells, c: Number of repetitive cells.

**Table 6:** Edge deployment results.

Model	PC		RASPBERRY PI 3 Model B+	
	Peak Memory (MB)	Inference Time (ms)	Peak Memory (MB)	Inference Time (ms)
Micro-ACDNet	5.25	0.98	5.17	44.31
ESC-NAS	4.78	1.32	4.76	57.33

**Table 7:** Related studies comparison for FSC22 dataset.

Model	Parameter Count (M)	Size (kB)	CV Score (%)
Micro-ACDNet [38]	0.120	484	85.64
HW-NAS [17]	0.028	230	83.05
ESC-NAS	0.092	365	85.78 ± 0.39

**Table 8:** Model performance comparison with benchmark raw audio classification models.

Dataset	Model		
	Micro-ACDNet	Micro-AclNet	ESC-NAS
FSC22	85.64	-	85.78 ± 0.39
US8K	78.28	75.80	81.25 ± 2.47
ESC-10	96.25	94.00	96.25 ± 0.22
ESC-50	83.65	80.05	81.00 ± 1.35

## Data Availability

FSC22 dataset: https://ieee-dataport.org/documents/fsc22-dataset (accessed on 6 May 2024). UrbanSound8K: https://urbansounddataset.weebly.com/urbansound8k.html (accessed on 6 May 2024). ESC-50: https://github.com/karolpiczak/ESC-50 (accessed on 6 May 2024). GitHub Repository: https://github.com/Neural-Dreamers/ESC-NAS (accessed on 6 May 2024).
